# Reverse-Bias and Temperature Behaviors of Perovskite Solar Cells at Extended Voltage Range

**DOI:** 10.1021/acsaem.1c03206

**Published:** 2022-02-17

**Authors:** Leyla Najafi, Sebastiano Bellani, Luca Gabatel, Marilena Isabella Zappia, Aldo Di Carlo, Francesco Bonaccorso

**Affiliations:** † BeDimensional S.p.A., Via Lungotorrente Secca 30R, 16163 Genova, Italy; ‡ CHOSE—Centre for Hybrid and Organic Solar Energy, University of Rome Tor Vergata, via del Politecnico 1, 00133 Rome, Italy; ∥ Istituto di Struttura della Materia, CNR-ISM, Via del Fosso del Cavaliere 100, 00133 Rome, Italy; ⊥ Graphene Laboratories, Istituto Italiano di Tecnologia, Via Morego 30, 16163 Genova, Italy

**Keywords:** perovskite solar cells, reverse bias, hot-spots, temperature, infrared thermal imaging

## Abstract

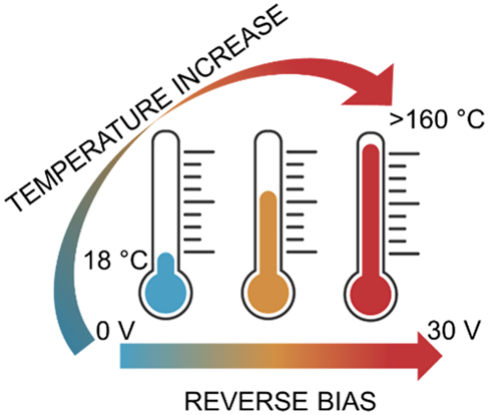

Perovskite solar cells have reached certified power conversion efficiency over 25%, enabling the realization of efficient large-area modules and even solar farms. It is therefore essential to deal with technical aspects, including the reverse-bias operation and hot-spot effects, which are crucial for the practical implementation of any photovoltaic technology. Here, we analyze the reverse bias (from 2.5 to 30 V) and temperature behavior of mesoscopic cells through infrared thermal imaging coupled with current density measurements. We show that the occurrence of local heating (hot-spots) and arc faults, caused by local shunts, must be considered during cell and module designing.

Metal halide perovskite solar cells (PSCs) represent a research hot-spot among various photovoltaic (PV) technologies due to their outstanding certified record-high power conversion efficiency (PCE) up to 25.5%, which competes with market-dominating polycrystalline Si solar cells.^1^ Recent efforts demonstrated the possibility to scale-up the PSC technology at a wafer scale, reporting a perovskite solar module with certified PCEs above 20%.^2^ These module PCE values promise to bridge the performance gap between the laboratory-scale and the practical large-area device concepts. As a striking example, an autonomous graphene-based perovskite solar farm system with a total output of 261 W peak (W_p_) has been launched last year in Crete, operating for a full year and showing the potential to lower the levelized cost of energy to less than 0.1 €/W_p_.^3^ Therefore, it is crucial to move the attention of researchers and engineers onto technological aspects related to the *real-world* functioning of entire PV systems, including design features of solar cells/modules, e.g., tilting angle, mounting and tracking systems, inverters, cell assembly, and module arrangement. Even more, the optimization of a solar farm design must evaluate the effects of shading and mismatch between solar cell performances on the plant reliability and safety.^4^ In fact, both shading and performance mismatch cause reverse biases across the shaded or less performant solar cells, which can result in module performance hysteresis,^5,6^ localized heating,^7^ and even hot-spot phenomena and other irreversible degradation effects.^7^ Although these technical aspects are crucial to secure reliable PV performances, as well as permits, licenses, and financing, they are still almost disregarded for PSC technology, probably because of its infancy stage compared to commercially available PV technologies. To the best of our knowledge, only sparse studies reported the reverse-bias behavior of PSCs,^5−13^ typically without elucidating the effect of reverse biases above a few volts,^14^ which, as discussed hereafter, can easily occur in module configurations (depending on the serial and/or parallel cell interconnections, as well as the use and choice of bypass diodes). During the revision of this work, a recent study associated the appearance of “sparkling” hot-spots to band bending and tunnelling current density caused by ion accumulation, and such effects are particularly pronounced in the presence of bulges in perovskite films associated with defective PbI_2_ clusters.^11^ It was noticed that the emergence of hot-spots is mitigated in high-PCE devices, which means that such devices can withstand high reverse biases (>3 V) before showing hot-spot-induced degradation.^11^ It was also shown that hot-spots are associated with transient current peaks, which is consistent with previous findings.^5^ These results suggest that very high (absolute) current densities may be locally reached over subsecond time scale. Another recent study on the reverse-bias behavior of PSCs with carbon-based electrodes extended the analysis at reverse biases exceeding 9 V.^12^ In addition, it was shown that the reverse-bias stability of such cells was superior compared to those based on metallic electrodes because of the lack of metal-induced degradation mechanisms (metal ion migration and even electrode melting at elevated temperatures).^12^ Nevertheless, it is still crucial to provide an in-depth understanding of the reverse-bias behavior of PSC with metallic electrodes, which are currently the most efficient PSC architecture, extending the analysis above a few volts (up to more than 10 V). Moreover, the analysis of PSC behavior at such reverse biases can serve as an accelerating aging test, which provides an approximate evaluation of the reliability of the devices in the presence of electric biases (that trigger ion migration/charge accumulation leading to thermally activated traps^15^ or detrimental parasitic electrochemical reactions^13^)^14^ or local defects/malfunctions, such as microstructural failures (e.g., pinholes in large-area perovskite films^16^ and ion-migration-induced conductive channels,^17^ which both cause a spatially confined reduction of the shunt resistance). Noteworthy, no ISOS standards have been reported for the evaluation of PSC stability under reverse bias stresses, even though a consensus has been reached on how to precondition devices before to assess PCE performance in order to exclude such bias-related effects on the device operation.^14^ Even more, only a few works analyzed experimentally the hot-spot effects in PSCs and perovskite solar modules,^8−10,12^ but, except for ref (12), their analysis was generally limited to maximum reverse bias of a few volts (e.g., −3 V) in single solar cells^9,11^ or at open circuit voltage (*V*_oc_) in solar modules.^10^ Meanwhile, a theoretical model for simulating the temperature of PSCs has been proposed in ref (18). However, this model does not describe the temperature behavior in the presence of device damages, which can even exacerbate the whole solar module degradation.

In this work, we report an experimental characterization of the reverse bias and temperature behavior of prototypical mesoscopic PSC configurations through reverse current density measurements–coupled infrared (IR) thermal imaging, aiming to elucidate the heating and degradation effects that are likely to occur under outdoor operation in the presence of shading and performance mismatch among the cells composing solar modules. Our data show intriguing effects, indicating that unsuitable PSC configurations can lead to abrupt temperature changes and even arc faults when reverse biases exceed a few (∼5) volts. The PSC configurations selected for the present study were prototypical mesoscopic PSCs based on TiO_2_ electron transporting layers (ETLs), spiro-OMeTAD hole transporting layer (HTL), and triple cation perovskite active layer. In particular, we focused on the following three device structures (Figure 1a,c): FTO/m-TiO_2_/perovskite/spiro-OMeTAD/Au (PSC-A), FTO/c-TiO_2_/m-TiO_2_/perovskite/spiro-OMeTAD/Au (PSC-B), FTO/c-TiO_2_/Li-doped m-TiO_2_/perovskite/spiro-OMeTAD/Au (PSC-C). The approximate thicknesses (provided by suppliers or measured through contact profilometry) of FTO, c-TiO_2_, m-TiO_2_ (or Li-doped m-TiO_2_), perovskite, spiro-OMeTAD, and Au layers are ∼500, ∼25, ∼150, ∼450, ∼250, and ∼85 nm, respectively. First, the comparison between PSC-A and PSC-B allows us to elucidate the role of the compact ETL in mesoscopic PSCs in limiting the degradation caused by their reverse polarization operation. Second, the comparison between PSC-B and PSC-A directly enables the understanding of the effect of Li-salt-doping of m-TiO_2_, which has been reported for the realization of hysteresis-free devices,^19^ on the reverse-bias and temperature behavior of PSCs. Figure 1d shows the layout used for the devices, whose active area is designated by a 1.67 cm × 0.6 cm strip. This layout was chosen to be compatible with the surface temperature monitoring through IR thermal imaging. It also allows us to reveal possible consequence of trivial geometrical asymmetries on the behavior of the device temperature as a function of the reverse bias. A photograph of a representative device (PSC-C) before the characterization of its reverse-bias behavior is shown in Supporting Information Figure S1.

**Figure 1:**
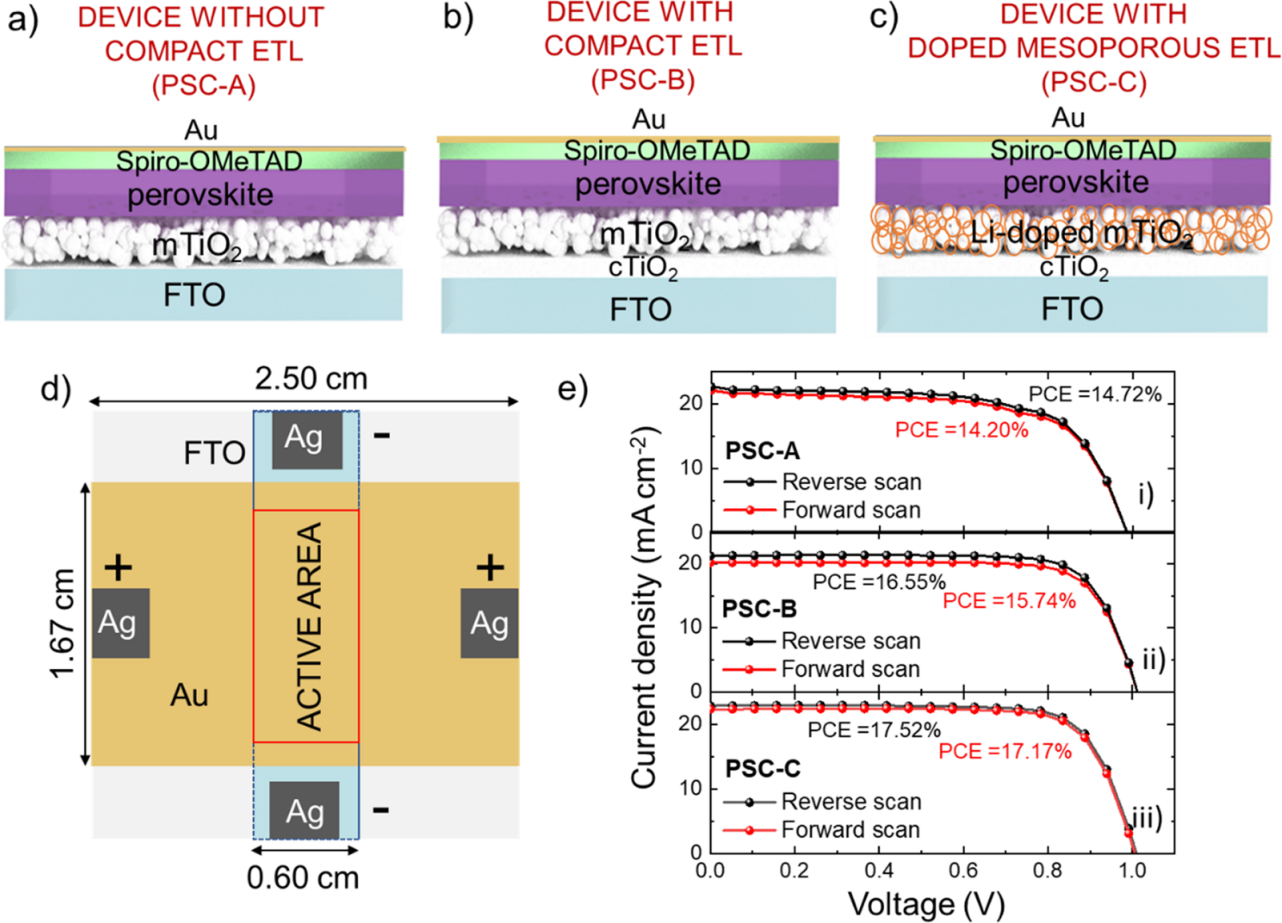
Sketches of the three mesoscopic PSC configurations investigated in this work: (a) FTO/m-TiO_2_/perovskite/spiro-OMeTAD/Au (PSC-A), (b) FTO/c-TiO_2_/m-TiO_2_/perovskite/spiro-OMeTAD/Au (PSC-B), and (c) FTO/c-TiO_2_/Li-doped m-TiO_2_/perovskite/spiro-OMeTAD/Au (PSC-C). (d) Layout of the electrodes used for the investigated PSCs, designating an active area of 1 cm^2^. (e) *J*–*V* curves measured for PSC-A, PSC-B, and PSC-C (replicas 1) in both reverse and forward voltage scan modes.

Figure 1e shows the current density vs voltage (*J*–*V*) curves measured for representative devices for each typology in both reverse and voltage scan modes. The PV characteristics, i.e., short circuit density (*J*_sc_), *V*_oc_, fill factor (FF), and PCE of the cells are summarized in Table S1. In agreement with previous studies,^20,21^ the absence of c-TiO_2_ causes severe charge recombination issues at the perovskite/FTO interface, leading to the lowest PCE of 14.72% (reverse scan) for PSC-A. Meanwhile, the Li-salt-doping of m-TiO_2_ lowers the conduction band edge of TiO_2_, assisting the electron injection and transport in the m-TiO_2_.^19^ Consequently, as expected from previous works,^19^ PSC-C showed a significant PCE increase and hysteresis reduction compared to PSC-B, reaching a PCE higher than 17% in both forward and reverse scan modes.

The reverse-bias and temperature behaviors of the three PSC configurations were investigated by scanning the voltage from 0 to −30 V, using a voltage-step protocol during which we measured the current density at a fixed voltage for 15 s (after a 15 s period of unbiased condition) while monitoring the temperature onto one side (rear or front) of the device, as depicted in Figure 2a. We point out that the reverse biases here analyzed are typically not considered when characterizing PSCs. However, cell reverse polarizations of a few and even up to tens of volts is likely to occur in solar modules because of partial shading and mismatch of the performance among the cells composing the module itself. As a striking example, when one cell in a serial module configuration is shaded or faulty, such a cell is forced into a maximum reverse bias (|*V*_REV_|_max_) determined by (1) its electrical breakdown (EB), i.e., (|*V*_REV_|_max_) ∼ electrical breakdown voltage (*V*_EB_) or, alternatively, (2) (in a prototypical serial module configuration) by the number of cells in the string (*n*), their *V*_oc_, and the forward voltage (*V*_f_) of the bypass diode used in the solar module, i.e.,^22^ |*V*_REV_|_max_ ≤ (∑_*i*=1_^*n*–1^*V*_oc,*i*_ + *V*_f_), in which *i* is the summation index. In the first case, the *V*_EB_ of the cell can limit the |*V*_REV_|_max_ to a maximum of a few volts, being a current density equal to those of the well-operating cell immediately delivered by the reverse-biased cell. However, the EB of the cell intrinsically reflects irreversible degradation effects, which may be local in nature (e.g., occurring in a defective spot, such as pinholes) and correspond to a severe local heating (well above 100 °C, as shown hereafter). A recent work associated the presence of high reverse current densities (and related hot-spots) to the existence of PbI_2_-rich bulges in perovskite layer.^11^ This indicates the importance to realize high-quality perovskite films to minimize reverse-bias degradation effects of PSCs, which in turn may also withstand reverse biases above 3 V.^11^ In the second case, being the *V*_oc_ of PSCs often superior to 1 V (see Figure 1e(ii,iii)) and the *V*_f_ of the bypass diode inferior to 1 V (smart bypass diodes with a low *V*_f_ of 26 mV are proposed by industries and companies), *n* equal to 10 already results in a reverse polarization of ca. 10 V. Noteworthily, such *n* values can be easily found in the most common perovskite solar module configurations, i.e., the so-called “mini-modules”, which consist of multiple PSCs connected in series to minimize the power losses originated by the electrical resistance of non-metallized large-area transparent electrodes. Thus, in the presence of high *V*_EB_ (which may prospectively be associated with high-quality perovskite films^11^), a reverse polarization of tens of volts could occur when such mini-modules are in turn connected in series for the realization of an entire solar panel with a m^2^-scale size (such as those of typical 72 cell polycrystalline Si solar panels, i.e., 164.0 cm (length) × 99.2 cm (width) = 1.63 m^2^). Even if the EB is not instantaneously reached, reverse biases can cause significant performance hysteresis,^5^ while triggering irreversible degradation effects (e.g., ion migration/charge accumulation-induced thermally activated traps/parasitic electrochemical reactions)^12,13,15^ that progressively lead to the cell EB with enormous dissipated power. Figure 2b shows the mean reverse current density measured at each reverse bias for three different device replicas (labeled as 1, 2, and 3) to ensure the reproducibility of the observed data trends. The inset panel reports the specific resistance (here defined as the ratio between the voltage and the mean reverse current density) measured for representative PSC configurations. In general, the reverse current densities were significantly pronounced in the absence of cTiO_2_ ETL (PSC-A), reaching values of tens of mA cm^–2^ at 2.5 V reverse bias and hundreds of mA cm^–2^ at reverse biases equal to or higher than 5 V. Although with certain variations from sample to sample, the Li-doping of mTiO_2_ (PSC-C) slightly increased the reverse current densities compared to those of the cell with undoped m-TiO_2_ (i.e., PSC-B). Contrary to PSC-A, both PSC-B and PSC-C started to exhibit relevant reverse current densities (on the order of tens of mA cm^–2^) at reverse biases equal to or higher than 7.5 V. The drop of the specific resistance of the cells, visualized in logarithmic scale in the inset of Figure 2b, can be attributed to the formation of local shunts,^12^ which can therefore occur easily in the cells without cTiO_2_ and may be also associated with Au ion migration effects.^12^ According to Ohm’s law, the power dissipated in the form of heat by the reverse-biased device (*P*) is given by the product of the current (*I*) and the voltage (*V*); i.e., *P* = *V* × *I*. Figure S2 reports the mean power density dissipated by each device as a function of the reverse bias, revealing maximum values as high as 9.41 W cm^–2^ for a PSC-A at 10 V reverse bias. To provide a qualitative idea of the relevance of these power density values, it is enough to consider that the heating power required for human thermal comfort for open-air space typically ranges from 0.1 to 0.2 W cm^–2^. To clearly correlate the dissipated power to a tangible temperature increase, Figure 2c reports the maximum temperature reached by representative devices over 15 s of reverse-bias operation for each reverse bias at the rear and front sides (replicas 1 and 2, respectively). Although it is critical to perform IR thermal imaging onto the rear side because of the reflectivity of the Au counter electrode, the recorded temperature nearby Au and the occurrence of Au film damage (e.g., crack formation or metal melting) still provide valuable information regarding the health state of the device. For the sake of clarity, the recorded temperature on Au is “apparent”, since it should be corrected by a factor equal to the inverse of Au emissivity (<0.1 for polished Au).^23^ In addition, the IR imaging from the rear side of the device is not affected by the thermal inertia (defined as the square root of the product of the material’s bulk thermal conductivity and volumetric heat capacity) of the 2.2 mm thick glass, which inevitably causes a nonmeasurable discrepancy between the maximum temperature achieved within the photoactive cell structure and the one of the outside glass. Consistently with the dissipated power data, PSC-A exhibited an abrupt increase of temperature at the rear side, up to more than 160 °C (maximum measurable temperature by our IR thermal imaging system). As shown in Figure 2d for replicas 2, the thermally induced mechanical stresses (i.e., thermal shocks) even led to the physical rupture of PSC-A in two pieces after applying reverse biases of 10 and 12.5 V in replica 2 (12.5 V in replica 3, see Figure 2b). Such radical deterioration was caused by a pronounced temperature gradient over time caused by hot-spots, which are attributed to local shunts.^12^ This disruptive mechanical deterioration was not observed in PSC-B and PSC-C, whose temperatures progressively increased (in some cases up to more than 160 °C; see replica 2 for PSC-B) with increasing the reverse bias until severe damages caused the electrical disconnection between cathode and anode through cracking and interface loosening of the ETL/perovskite/HTL structure. Such effects typically occurred at reverse biases between 15 and 25 V. Therefore, as shown in the photographs reported in Figure 2d, the entire active area of PSC-B and PSC-A was deteriorated after applying a reverse bias of 30 V, evidencing pronounced cracking/stripping/dissolution of the Au electrodes.

**Figure 2:**
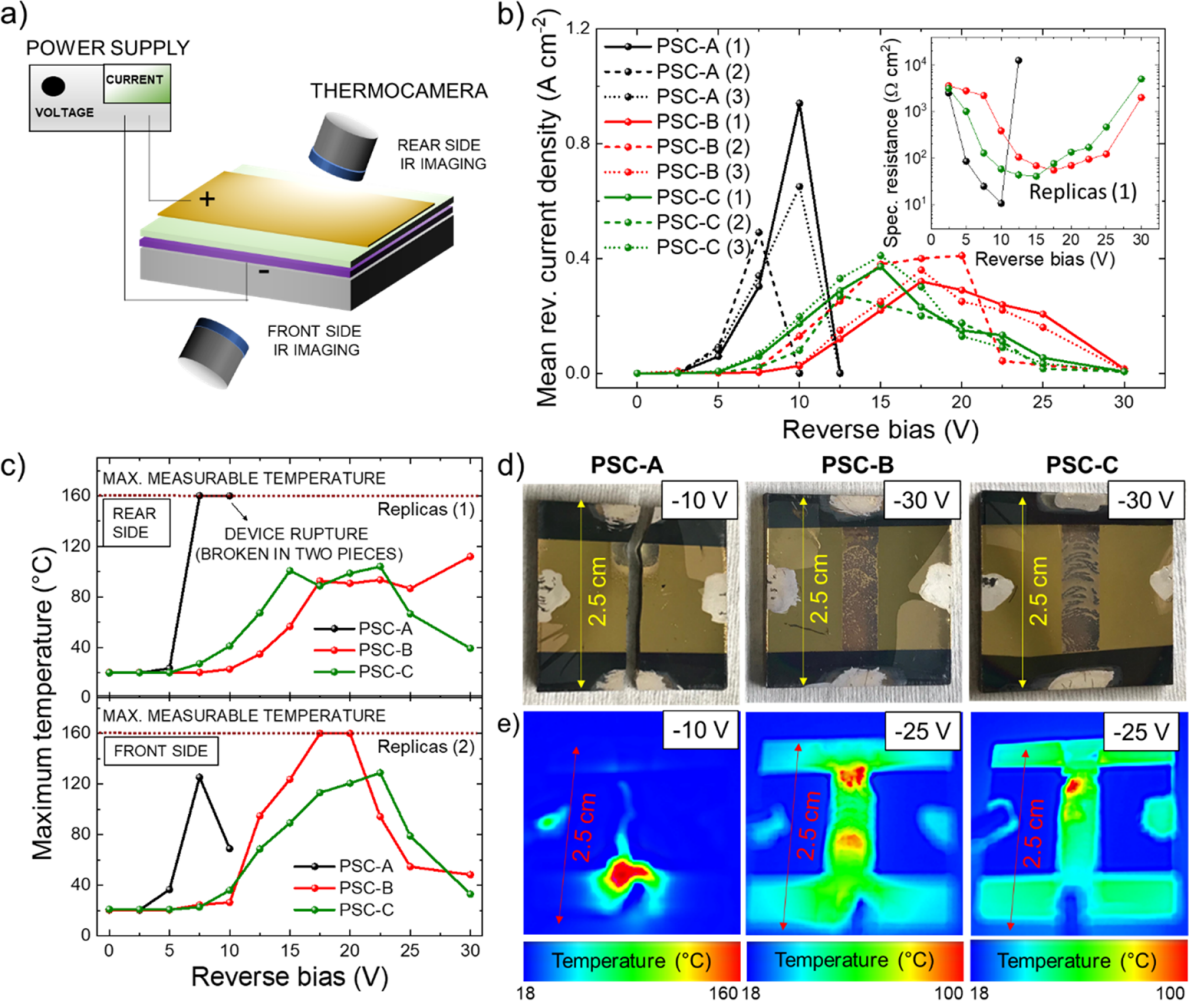
(a) Sketch of the experimental setup used to characterize the reverse-bias and temperature behaviors of the investigated PSCs, using a DC power supply unit and a thermal camera. The investigated devices were fabricated on 2.5 cm × 2.5 cm glass substrates, and their active area is 1 cm^2^ (designated by a 1.67 cm × 0.6 cm strip), as detailed in Figure 1d. (b) Mean reverse current density vs reverse-bias plots measured for the investigated devices. The inset shows the specific resistance (defined as the ratio between the voltage and the mean reverse current density) vs reverse bias for a representative device for each configuration (replicas 1). (c) Maximum temperature vs reverse bias plots for representative devices for each PSC configuration, in which the temperature was measured on the rear side (replicas 1) or front side (replicas 2). (d) Photographs of PSC-A, PSC-B, and PSC-C (replicas 1) after reverse-bias operation at 10 V for PSC-A and at 30 V for PSC-B and PSC-C. (e) Temperature maps for PSC-A, PSC-B, and PSC-C measured during reverse bias operation at 10 V for PSC-A and 30 V for PSC-B and PSC-C (replicas 1). The maps have been taken from the rear side of the cells at the time corresponding to the maximum temperature reached by the devices at the corresponding reverse bias.

Nevertheless, such reverse biases can cause uncontrolled sparks (i.e., arc faults), as shown in Video S1. Above the formation of filamentary shunts caused by metal ion migration,^12^ we observed that, once the device structure is significantly deteriorated, it loses the electrical connection between cathode and anode. Consequently, arc faults can be also ascribed to the presence/formation of pinholes or loose device interfaces (as those at the edges of the active area), resulting in spacings that cause the EB of the surrounding medium. Importantly, the occurrence of arc faults must be carefully considered to avoid fire accidents in PV systems.^24^Figure 2e shows the temperature maps of the investigated devices (replicas 1) at the maximum reverse bias reached by the devices at their rear side before the drop of their reverse current toward near-zero values (electrical disconnection). In PSC-A, the heat linearly propagated over the length of the active area, determining the fracture line of the device (see also Video S2). For PSC-B and PSC-C, the heating spread over the whole active area of the devices, which was finally completely degraded after the experiments. However, as shown in Figure S3 for a representative case (replica 1 of PSC-C), the initial integrity of the Au film in PSC-B and PSC-C away from the contact can provide an efficient thermally conducting pathway that distributes the heat over the whole device active area, which then completely deteriorates without reaching temperature gradients that cause the glass breaking, as instead observed in PSC-A. As previously discussed, the temperature detected over the Au electrode is “apparent” due to the low Au emissivity. Consistently, the highest temperatures recorded by our IR thermal imaging system were near the bottom border of the Au film, where the current is also collected through the underlying FTO electrode. The latter was electrically contacted from its bottom side. However, characteristic temperature maps measured for representative devices (replicas 3) at their front side (Figure S4) show that the maximum temperature in PSC-B and PSC-C is reached over the active area. As previously discussed, the thermal inertia of the 2.2 mm thick glass substrate causes a nonmeasurable discrepancy between the maximum temperature achieved within the photoactive cell structure and the one of the exposed substrate surface. Therefore, it is reasonable that the temperature reached in the active materials of the devices is significantly higher than those measured by our IR thermal imaging system at the front side of the cell, as also proved by rear–front analyses (Figure 2e). According to a recent study,^12^ hot-spots generated by the passage of high current in filamentary shunt can cause the thermal decomposition of the perovskite into PbI_2_. Since the latter is less conductive, the surrounding areas of perovskite can become the path of least resistance, which in turn will be degraded by the heating induced by the increase of the current. These considerations can explain the movement of hot-spots during our tests, as visualized by our IR thermal imaging over time (Figure S5), during which the reverse bias was progressively increased from 2.5 to 20 V, using 2.5 V step with a duration of 15 s. Of course, if defective shunts are present, degradation will first occur at those defects, since they represent the path of least resistance, facilitating the passage of the current.^12^

To further elucidate the effect of the reverse bias on the PV characteristics of the devices, Figure 3a compares the *J*–*V* curves of representative devices measured before and after a reverse bias of 2.5 V for 15 s, without waiting for possible performance recovering.^5,13^ Noteworthy, cell performance recovery after reverse biases up to 5 V, i.e., similar to those considered in Figure 3a, has been subject matter of important recent literature, to which we refer the readers for in-depth discussion.^5,13^ Even though no relevant reverse current density and temperature changes were recorded at reverse bias of 2.5 V for all of the investigated devices (see Figure 2c), all the devices showed a significant decrease of the PV performances, in agreement with previous studies.^5,13^ As summarized in Table S1, the most significant performance degradation was observed in PSC-A, which decreased the PCE from 14.72 to 4.17% (reverse scan). Recent theoretical modeling of perovskite solar modules suggested that, if degradation problems during breakdown regime are solved, PSCs with low *V*_EB_ may be an ideal solution to solve the issues related to partial shading or performance mismatch of solar panels, being that the PSCs themselves act as bypass diodes.^7^ However, as also highlighted in ref (7), the absence of deleterious degradation under breakdown regime still hardly reflects the real operation conditions (as shown here above, in particular for PSC-A). In our case, PSC-C displayed the highest *V*_EB_, while undergoing a limited degradation compared to PSC-B, experiencing a PCE drop from 17.52 to 13.30% (reverse scan). Even though it has been demonstrated that the performance of PSC after reverse voltage inferior to a few volts can be recovered during minute/hour time scale at maximum power point operation,^5,13^ the permanent reduction in power output can keep the cell pinned in reverse bias when interconnected in series within a solar module.^8^ This effect may progressively trigger irreversible degradation pathways at local scale and must be therefore carefully considered when designing both the cell structure and the cell interconnection in perovskite solar modules.

**Figure 3:**
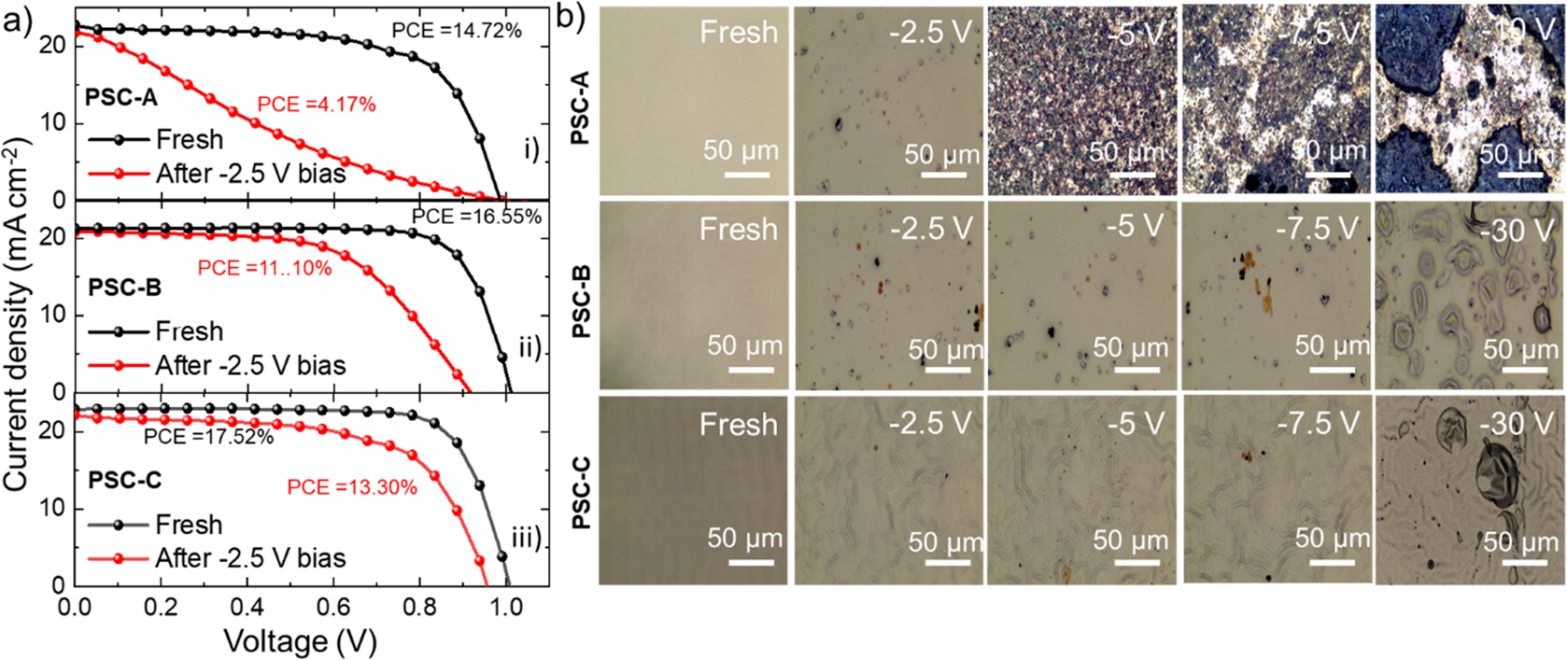
(a) Comparison between the *J*–*V* curves measured for the investigated devices (replicas 1) before and after 15 s of reverse polarization at −2.5 V. (b) Optical microscopy images of the rear side (Au electrode) of the investigated PSCs (replicas 1) before (0 V) and after various reverse bias polarizations.

Lastly, optical microscopy imaging of the rear side of the cells (Figure 3b) evidenced physical degradation already after a reverse bias of 2.5 V. By increasing the reverse bias above 2.5 V, the degradation significantly increased, causing cracking and stripping of the Au electrode in PSC-A, while the Au electrodes of PSC-B and PSC-C experienced the formation of crater-like spots, likely attributed to local hot-spots, before showing Au delamination/stripping/melting phenomena at reverse biases between 15 and 25 V.

In summary, our results shed light on the reverse-bias behavior of prototypical mesoscopic PSC configurations, up to voltages of tens of volts, which may occur in cells within solar panels. We point out that such polarization conditions can serve as an accelerating test to evaluate the reliability of device configurations, whose large-area function likely includes local defects/malfunctions (e.g., pinholes in large-area perovskite films and ion-migration-induced conductive channels) that reflect low-shunt resistance channels. The absence of a compact ETL (i.e., c-TiO_2_) accelerates the reverse bias-induced temperature increase in such a way that the physical rupture of the device can also occur. The Li-salt-doping of the m-TiO_2_ does not significantly alter the reverse bias operation of the devices. Therefore, it is confirmed as a good strategy to improve the PSC performance. In all devices, the local heating and the presence of arc faults, associated with local shunts induced by metal ion migration effects, can cause the stripping/erosion/melting of the Au electrodes. Regarding this point, recent studies showed that PSCs with carbon-based electrodes can mitigate the degradation of the cells under high reverse biases because of the lack of metal-induced degradation mechanisms (electrode melting and metal ion diffusion at elevated temperatures).^12^ Nevertheless, our results also remark on the importance of considering technical aspects related to temperature changes and hot-spot effects when designing the PSC structure, as well as their interconnection within solar modules, to ensure reliable and safe operation of the final perovskite PV plants. Importantly, our work indicates that the charge transport layer engineering represents an effective strategy to increase not only the PV performance of the PSCs^25−27^ and perovskite solar modules^25,28^ but also their device stability and reliability during practical operation.^25,29^ Moreover, the serial module configurations adopted for the realization of wafer-scale perovskite PV systems is intrinsically subjected to the reverse bias effects in the presence of cell faults or cell performance mismatch. The assembly of large-area PSCs in solar module through hybrid series/parallel or parallel configurations may overcome such limitations,^3,30^ even though large currents increase the resistive losses when extracting current from the module to the junction box.^7^ Meanwhile, the realization of wafer-area PSCs (preferred in parallel module configurations) is still challenging and can even lower the cell *V*_EB_ in the presence of unavoidable nano-/microscale defects, leading to local overheating-induced irreversible degradation in nonreverse conditions. Therefore, these considerations, together with the results shown above, represent a stimulus for the perovskite PV community to focus the attention also to technical aspects related to large-scale solar panels and farms, until now almost disregarded. Our results, together with recent theoretical modeling of both single-junction PSCs and perovskite-based tandem cells,^7^ can also spur the establishment of new ISOS standards for the evaluation of PSC stability under reverse biases or during their operation in module assemblies. Further studies on the reverse bias behavior of perovskite PV technologies are therefore expected (and foreseen by our group) on more advanced PSC configurations and wafer-scale perovskite solar modules.

## Supporting Information Available

Video S1 showing effects of reverse biases on device (MOV)
Video S2 showing heat linearly propagated over length of active area (MOV)
Device fabrication and characterization methods; photovoltaic characteristics of the devices; mean dissipated power density power analysis; supplementary temperature maps of devices during operation (PDF)

## Supplementary Material

ae1c03206_si_001.mov

ae1c03206_si_002.mov
