# Supplementary material for: Reverse-Bias and Temperature Behaviors of Perovskite Solar Cells at Extended Voltage Range
Source: ACS Appl Energy Mater. 2022 Feb 17;5(2):1378–84. doi: 10.1021/acsaem.1c03206 (PMC8889533; doi:10.1021/acsaem.1c03206)
Supplement: Supplementary file 3 — ae1c03206_si_003.pdf [file ae1c03206_si_003.pdf]

Supporting Information

Reverse-bias and temperature behaviour of perovskite solar cells at extended voltage range

Leyla Najafi,[1‡] Sebastiano Bellani,[1,‡,] Luca Gabatel,[1] Marilena Isabella Zappia,[1] Aldo Di Carlo[2,3,4] and Francesco Bonaccorso[1,5]**

[1] BeDimensional S.p.A., Via Lungotorrente Secca 30R, 16163 Genova, Italy

[2] CHOSE – Centre for Hybrid and Organic Solar Energy, University of Rome Tor Vergata, via del Politecnico 1, 00133, Rome, Italy

[3] LASE – Laboratory for Advanced Solar Energy, National University of Science and Technology MISiS, Moscow 119049, Leninsky, Ave. 6, Russia

[4] Istituto di Struttura della Materia, CNR-ISM, Via del Fosso del Cavaliere 100, 00133, Rome, Italy

[5] Graphene Labs, Istituto Italiano di Tecnologia, Via Morego 30, 16163 Genova, Italy

AUTHOR INFORMATION

ǂ These two authors equally contributed to this work

Corresponding Author

*e-mail: s.bellani@bedimensional.it; f.bonaccorso@bedimensional.it

METHODS

Device fabrication. Etched fluorine doped tin oxide (FTO) substrates (25 mm×25 mm×2.2 mm, 6-8 Ω sq^{-1}, BIOTAIN HONG KONG CO., LIMITED) were cleaned using a 2% Hellmanex cleaning solution diluted with water, in which they were sonicated for 15 min. Subsequently, the substrates were rinsed with deionized water and sonicated in isopropanol for 15 min and in acetone for 15 min. Afterwards, the substrates were dried and cleaned from organic residuals by oxygen plasma treatment using an inductively coupled radio frequency (13.56 MHz) reactor (Colibrì, GAMBETTI Kenologia Srl) at a power of 200 W and a process pressure of 40 Pa (background gas pressure of 0.2 Pa) for 10 min. The so-prepared substrates were transferred to a hot plate and heated up to 450 ˚C within 30 min. Then, the substrates were kept at 450 ˚C for 20 min, after which a compact TiO_2 (c-TiO_2) layer was deposited by spray pyrolysis method. The sprayed solution

contained 0.4 mL of acetylacetone (Sigma-Aldrich) and 0.6 mL of titanium diisopropoxide bis(acetylacetonate) (Sigma-Aldrich) in 9 mL of ethanol (≥99.8%, Sigma Aldrich). After spraying, the samples were left for 10 min at 450 ˚C and then cooled down to room temperature. The thickness of the resulting c-TiO_2 layer was ~25 nm, as measured by contact profilometry (XP-2 Profiler, Ambios). The mesoporous TiO_2 (m-TiO_2) layer was deposited by spin coating 150 mg ml^{-1} of 30 N-RD TiO_2 paste (Greatcell Solar Materials) diluted in ethanol (≥99.8%, Sigma Aldrich). Before being used, the diluted paste was homogenized by continuous magnetic stirring overnight. The resulting paste was spin coated using a one-step protocol with a rotational speed of 4000 rpm (1000 rpm s^{-1} acceleration rate) for 10 s to deposit the m-TiO_2 layer. Immediately after spinning the paste, the deposited layer were dried at 100 ˚C for 10 min. Subsequently, the m-TiO_2 layer was sintered at 460 °C for 30 min and then cooled down to room temperature. The thickness of the resulting m-TiO_2 layer was ~150 nm, as measured by contact profilometry (XP-2 Profiler, Ambios). For the samples named PSC-A, the m-TiO_2 layer was directly deposited atop the FTO substrate without c-TiO_2 layer. For the samples named PSC-B, the m-TiO_2 layer was instead deposited atop the c-TiO_2. Lastly, the samples named PSC-C were produced by doping the m-TiO_2 layer with Li salt. More in detail, a solution of 10 mg mL^{-1} bis(trifluoromethane)sulfonamide

lithium salt (Li-TFSI) (99.95% trace metals basis, Sigma Aldrich) in acetonitrile (ACS reagent, ≥99.5%, Sigma Aldrich) was spin coated onto the m-TiO_2 layer with a rotational speed of 3000 rpm (1000 rpm s^{-1} acceleration rate) for 10 s. Afterwards, all the samples were sintered at 460 ℃ for 30 min and then cooled down to 150 ˚C. The samples were then moved into a glovebox and cooled down to room temperature. The perovskite layer was prepared from a precursor solution of PbI_2 (TCI America) (1.19 M), formamidinium iodide (Greatcell Solar Materials) (FAI) (1.04 M), methylammonium bromide (MABr) (Greatcell Solar Materials) (0.15 M), $PbBr_2$ (TCI America) (0.15 M) and CsI (abcr GmbH) (0.1 M) in anhydrous N,N-Dimethylformamide (DMF):Dimethyl sulfoxide (DMSO) (4:1 vol:vol) (≥99.9%, Sigma Aldrich). The so-prepared solution was spin coated onto m-TiO_2 (or Li-doped m-TiO_2) using a two-step protocol (first step: 1000 rpm and 200 rpm/s for 10s; second step: 4000 rpm and 2000 rpm/s for 30 s. During the second step, 110 μL of chlorobenzene (anhydrous, 99.8% Sigma Aldrich), was dropped in the middle of substrate 5 s prior to the end of the program. After the spin coating step, the samples were annealed at 100˚C for 60 min. The thickness of the resulting perovskite layer was ~450 nm, as measured by contact profilometry (XP-2 Profiler, Ambios). The 2,2′,7,7′-tetrakis-(N,N-di-4-methoxyphenylamino)-9,9′spirobifluorene (spiro-OMeTAD) layer was subsequently deposited onto the perovskite by spin

coating a 70 mM solution of spiro-OMeTAD (Borun New Material Technology) in chlorobenzene (anhydrous, 99.8% Sigma Aldrich) doped with (Li-TFSI) (Sigma-Aldrich), tris(2-(1H-pyrazol-1-yl)-4-tert-butylpyridine)- cobalt(III) tris(bis(trifluoromethylsulfonyl) imide) (FK209) (Sigma-Aldrich) and 4-tert-Butylpyridine (TBP) (Sigma-Aldrich) with molar ratios of 0.5, 0.03 and 3.3 respectively. 45 μL of doped spiro-OMeTAD solution was deposited on top of the perovskite film, while spinning at 4000 rpm for 20 s (2000 rpm s^{-1} acceleration rate). The thickness of the resulting spiro-OMeTAD layer was ~250 nm, as measured by contact profilometry (XP-2 Profiler, Ambios). The devices were completed by depositing 85 nm of Au through high-vacuum (10^{-6} Pa) thermal evaporation using a thickness deposition rate of 0.3 Å s^{-1} for the first 20 nm and 1 Å s^{-1} up to 85 nm.

Device characterization. To evaluate the photovoltaic performance of the solar cells, the current density-voltage (J-V) curves were recorded in air using a Keithley 2400 source meter under 100 mW cm^{-2} (AM1.5G) illumination, provided by a Oriel Sol3A class AAA solar simulator (Newport). The light intensity was calibrated using a Newport 91150V reference cell. The reverse bias and temperature behaviour of the solar cells were investigated by scanning the voltage from 0 V to -30 V, using a voltage-step protocol during which we measured the current density at a

fixed voltage for 15 s (after a 15 s period of unbiased condition) while monitoring the temperature onto the rear side or front side of the device. The reverse bias was applied using a bench DC power supply (EL302RT, Aim-TTi), while the temperature was monitored through IR thermal imaging with a thermal camera (A655sc, FLIR) placed at ~40 cm from the sample surface. The thermal camera was controlled with FLIR's own software (Temperature FLIR ResearchIR Max software), which was also used to process the temperature data. Photographs of the entire devices were acquired with a smartphone digital photocamera. Optical images of portions of device's surface were acquired with an Leyca optical microscope.

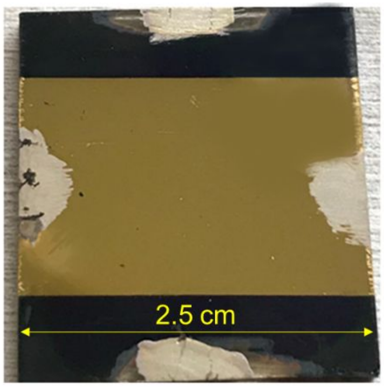

Figure S1. Photograph of a representative device (PSC-C) before the characterization of its reverse bias behaviour. The device was fabricated on a 2.5 cm × 2.5 cm glass substrate, and its active area is 1 cm^2 (designated by a 1.67 cm × 0.6 cm strip), as detailed in **Figure 1d** of the main text.

Table S1. Photovoltaic characteristics measured in reverse and forward voltage scan modes for the investigated mesoscopic PSCs.

Device configuration	Voltage scan mode	J_{sc} (mA cm^{-2})	V_{oc} (V)	FF (%)	PCE (%)
**PSC-A**	**Reverse**	22.71	0.998	65.55	14.72
	**Forward**	22.10	0.988	64.99	14.20
**PSC-B**	**Reverse**	21.23	1.011	77.07	16.55
	**Forward**	20.17	1.011	77.14	15.74
**PSC-C**	**Reverse**	22.9	1.009	76.21	17.62
	**Forward**	22.37	1.005	76.35	17.17
**PSC-A (after 15 s at -2.5 V)**	**Reverse**	21.81	0.996	19.17	4.17
	**Forward**	21.67	0.987	16.94	3.63
**PSC-B (after 15 s at -2.5 V)**	**Reverse**	20.86	0.918	57.96	11.10
	**Forward**	20.72	0.908	54.39	10.24
**PSC-C (after 15 s at -2.5 V)**	**Reverse**	22.102	0.956	62.89	13.3
	**Forward**	21.73	0.949	62.18	12.83

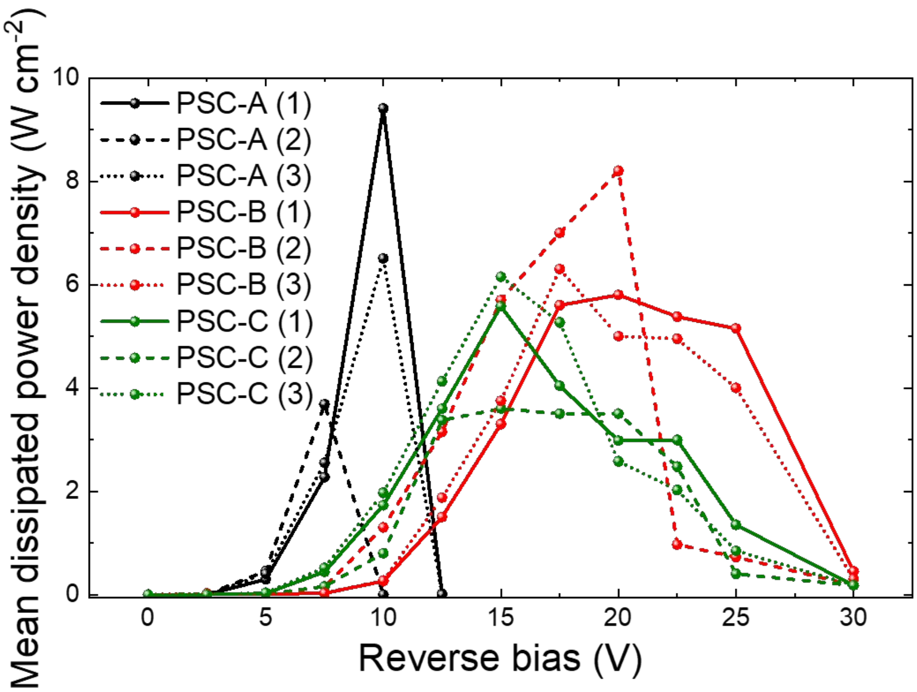

Figure S2. Mean dissipated power density mean power density *vs.* reverse bias plots measured for the investigated devices (three replicas for each device configuration).

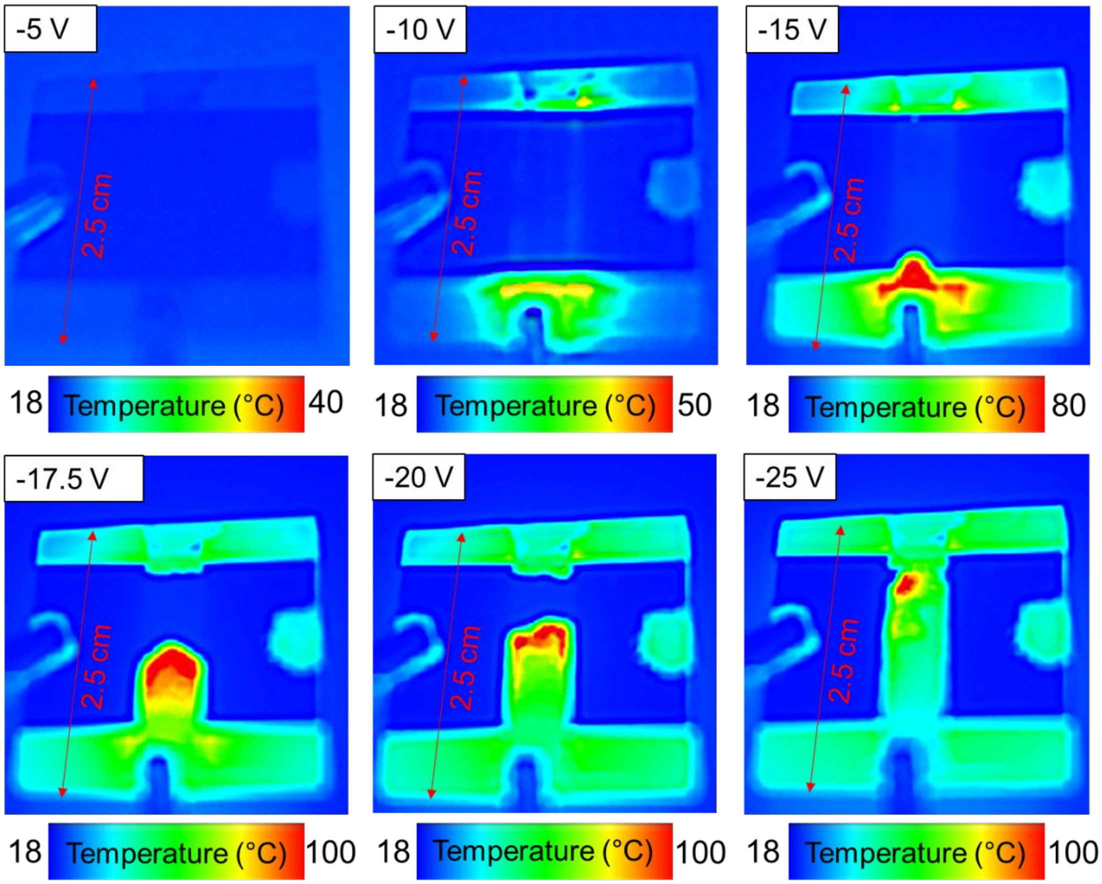

Figure S3. Temperature maps measured for a PSC-C (replica (1)) at the rear side during operation at increasing reverse biases, from 5 V to 25 V. The maps have been taken at the time corresponding to the maximum temperature reached by the device for each reverse bias condition. The investigated device was fabricated on a 2.5 cm × 2.5 cm glass substrate, and its active area is 1 cm^2 (designated by a 1.67 cm × 0.6 cm strip), as detailed in **Figure 1d** of the main text.

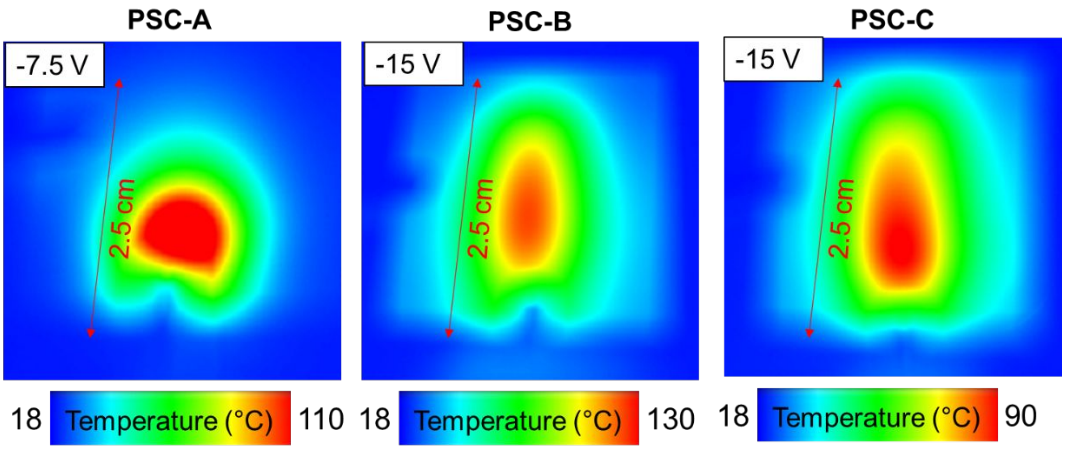

Figure S4. Temperature maps measured for representative devices (replicas (2)) at the front side during operation at reverse biases of 7.5 V for PSC-A and 15 V for PSC-B and PSC-C. The maps have been taken at the time corresponding to the maximum temperature reached by the devices for each reverse bias condition. The investigated devices were fabricated on 2.5 cm × 2.5 cm glass substrates, and their active area is 1 cm^2 (designated by a 1.67 cm × 0.6 cm strip), as detailed in **Figure 1d** of the main text.

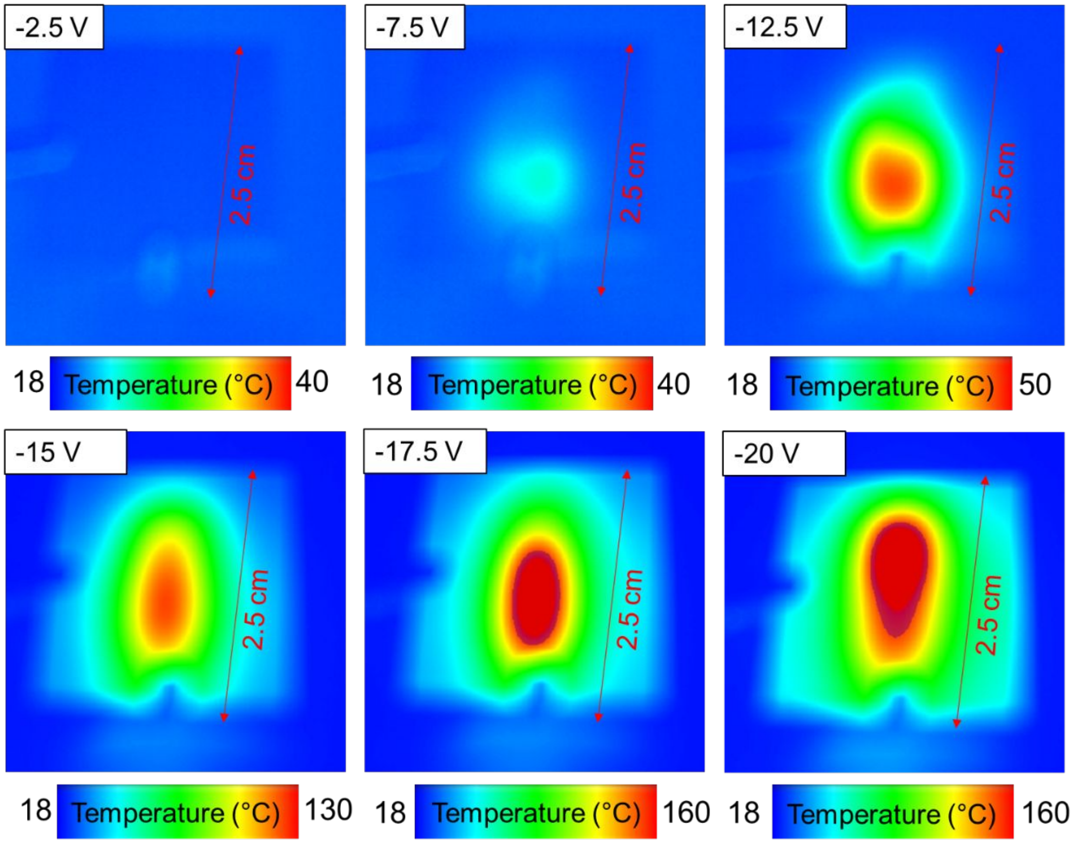

Figure S5. Temperature maps measured for a PSC-B (replica (2)) at the rear front side during operation at increasing reverse biases, from 2.5 V to 20 V. The maps have been taken at the time corresponding to the maximum temperature reached by the device for each reverse bias condition. The investigated device was fabricated on a 2.5 cm × 2.5 cm glass substrate, and its active area was 1 cm^2 (designated by a 1.67 cm × 0.6 cm strip), as detailed in **Figure 1d** of the main text.
